# Possible Functional Roles of Patellamides in the Ascidian-*Prochloron* Symbiosis

**DOI:** 10.3390/md20020119

**Published:** 2022-02-02

**Authors:** Philipp Baur, Michael Kühl, Peter Comba, Lars Behrendt

**Affiliations:** 1 Institute of Inorganic Chemistry, Heidelberg University, Im Neuenheimer Feld 270, 69120 Heidelberg, Germany; philipp.baur@aci.uni-heidelberg.de; 2 Max Planck School Matter to Life, 69120 Heidelberg, Germany; 3 Marine Biology Section, Department of Biology, University of Copenhagen, Strandpromenaden 5, DK-3000 Helsingør, Denmark; 4 Interdisciplinary Center of Scientific Computing, Heidelberg University, 69120 Heidelberg, Germany; 5 Department of Organismal Biology, Science for Life Laboratory, Uppsala University, Norbyvägen 18 A, 752 36 Uppsala, Sweden

**Keywords:** patellamide, cyanobactin, symbiosis, *Prochloron*, didemnid ascidians

## Abstract

Patellamides are highly bioactive compounds found along with other cyanobactins in the symbiosis between didemnid ascidians and the enigmatic cyanobacterium *Prochloron*. The biosynthetic pathway of patellamide synthesis is well understood, the relevant operons have been identified in the *Prochloron* genome and genes involved in patellamide synthesis are among the most highly transcribed cyanobacterial genes in hospite. However, a more detailed study of the in vivo dynamics of patellamides and their function in the ascidian-*Prochloron* symbiosis is complicated by the fact that *Prochloron* remains uncultivated despite numerous attempts since its discovery in 1975. A major challenge is to account for the highly dynamic microenvironmental conditions experienced by *Prochloron* in hospite, where light-dark cycles drive rapid shifts between hyperoxia and anoxia as well as pH variations from pH ~6 to ~10. Recently, work on patellamide analogues has pointed out a range of different catalytic functions of patellamide that could prove essential for the ascidian-*Prochloron* symbiosis and could be modulated by the strong microenvironmental dynamics. Here, we review fundamental properties of patellamides and their occurrence and dynamics in vitro and in vivo. We discuss possible functions of patellamides in the ascidian-*Prochloron* symbiosis and identify important knowledge gaps and needs for further experimental studies.

## 1. Introduction 

### Discovery of Prochloron and the Ascidian-Prochloron Symbiosis

**Figure 1:**
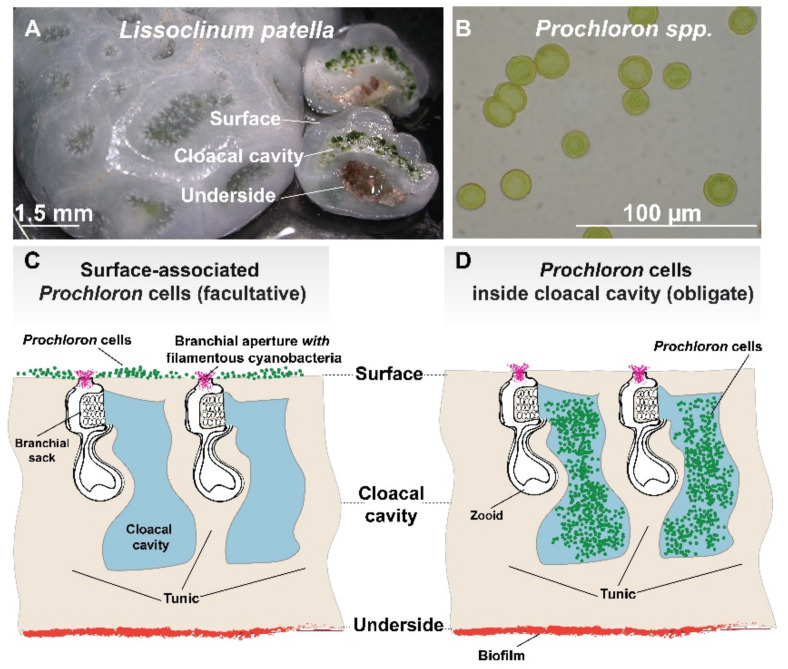
The photosymbiotic association between didemnid ascidians and the cyanobacterium *Prochloron*: (**A**) Image of the didemnid ascidian *Lissoclinum patella*, a common host for cyanobacterial *Prochloron* photosymbionts. Cross sections of *L. patella* (right side in image **A**) illustrate the distinct zonation of microbial biofilms occurring on (i) the surface of the animal, (ii) in its cloacal cavity and (iii) on its underside. (**B**) Single *Prochloron* cells extracted from *L. patella*. Note the cells’ deep green color that imbues the cloacal cavity of *L. patella* with the same color (see panel **A**). (**C**) A schematic overview of a didemnid ascidian in cross-section with surface-associated *Prochloron* cells. Animal zooids are embedded within a cartilaginous tunic consisting of proteins and cellulose-like carbohydrates. Zooids draw in seawater to capture particles for heterotrophic feeding. Animal waste products and excess water is excreted into the space surrounding the branchial sack as well as the cloacal cavity. Note the presence of *Prochloron* epibionts (in green) and filamentous cyanobacteria in the branchial aperture (in pink) on the surface of the ascidian. (**D**) A schematic overview of a didemnid ascidian in cross-section with *Prochloron* cells within its cloacal cavity. Note the dense packing of *Prochloron* cells within the cloacal cavity of the ascidian (e.g., *L. patella*) as well as the microbial biofilms occurring on the underside of the animal.

*Prochloron* are unicellular (~10–25 µm), spherical cyanobacteria with appressed thylakoids that engage in symbiosis with (sub)tropical didemnid ascidians (tunicates) and, less commonly, some marine invertebrates such as porifera and holothurians [1]. The ascidian-*Prochloron* symbiosis is the only known example of obligate photosymbiosis in chordates. While they can be found in seawater filtrates from coral reef waters, and may persist in a free-living state [2], *Prochloron* is mainly found colonizing the interior or exterior surface of didemnid ascidians. Didemnid ascidians are colonial filter feeding animals, where many individual zooids are embedded in a common tunic (also called test), which is a transparent or semitransparent matrix made from protein and cellulose-like material and sometimes contains calcareous spicules. The ascidian-*Prochloron* symbiosis has different manifestations [3,4] (Figure 1), where the cyanobacterium can either be considered (i) an obligate symbiont hosted intracellularly in tunic cells (e.g., in *Lissoclinum punctatum*) or, more commonly, colonizing the inner cloacal cavities (e.g., in *Lissoclinum patella*, *Diplosoma* spp.) and tunic (e.g., some *Didemnum* and *Trididemnum* species) of the ascidian, or (ii) facultatively associated, where *Prochloron* is more loosely bound in a biofilm on the external tunic surface (e.g., of *Didemnum candidum* and some non-didemnid ascidians).

The association of didemnid ascidians with unicellular phototrophs has been known for many years [5,6], and active photosynthesis by such cells in hospite was first reported in 1942 [7]. However, it was the pioneering work of Ralph Lewin that led to the discovery of *Prochloron* spp. as unique photosymbionts of didemnid ascidians. Here the species was initially described as *Synechocystis didemnii* and later renamed to *Prochloron didemni* [8]. When *Prochloron* was first described [9,10], it was the first example of a prokaryotic phototroph with chlorophyll *a* and *b*, and without any phycobiliproteins [11]. Together with other features of its photosynthetic apparatus such as appressed thylakoids, *Prochloron* was initially considered a missing link in chloroplast evolution and thought to represent an oxygenic phototrophic bacterium, from which green plastids had evolved. This inspired the name *Prochloron* and led to its classification as a prochlorophyte [12], a bacterial order that now also encompasses other cyanobacteria, i.e., *Prochlorococcus* and *Prochlorothrix*, with similar, unusual photopigmentation. With the advent of molecular ecology, it is now well established that prochlorophytes represent a different lineage among the cyanobacteria [13], and a direct evolutionary link between *Prochloron* and green plastids has not been established (e.g., [14]). 

Today, more than 45 years since its discovery in 1975, *Prochloron* remains uncultivated despite the fact that it is easy to collect and harvest large cell numbers from ascidians. Typically, such separated *Prochloron* cells can remain viable and photosynthetically active for a few days before becoming inactive and ultimately degraded by bacterial overgrowth. However, a recent report [15] described the successful maintenance of growing *Prochloron* cells for several months after initial separation from the ascidian *Lissoclinum patella*, reaching cell densities of up to 10^6^–10^7^ mL^−1^. Molecular analysis of the 16S *rRNA* gene and the gene coding for chlorophyll *a* oxygenase (*CAO*) in enrichment cultures showed high similarity to *Prochloron didemni*, and cells produced characteristic secondary metabolites such as patellamide E and ulithiacyclamide [16]. These promising reports indicate that stable long-term cultivation of *Prochloron* is within reach, which would be instrumental for more detailed studies of some of the many knowledge gaps in understanding the cell biology and the ecological and physiological role of bioactive secondary metabolites in *Prochloron* and their potential role in regulating the ascidian-*Prochloron* symbiosis. 

The importance of *Prochloron* for its host remains underexplored, but the fact that several behavioral and structural mechanisms have evolved for ensuring transmission of *Prochloron* cells to ascidian larvae as they leave the parent colony [3,17] points to an important role of *Prochloron* for ascidian fitness. In situ experiments on coral reefs, showed that limiting *Prochloron* photosynthesis (by shading) slowed the growth of the ascidian host [18], and a significant transfer of photosynthates from *Prochloron* to the ascidian host has been demonstrated [19]. This supply is estimated to cover up to 60% of the animal carbon demand (e.g., [20]), although it is argued that the contribution of *Prochloron* to host carbon metabolism is more variable [20,21]. Generally, the influence of the microbiome on the fitness and growth of the ascidian host has yet to be studied in more detail, as well as the actual transfer mechanisms for carbon, nutrients and secondary metabolites between *Prochloron* and its host, as they could be another important niche-shaping factor in the ascidian-*Prochloron* symbiosis.

*Prochloron* is able to synthesize various cyanobactins, which exhibit strong bioactivity and have been intensively studied with respect to their structural chemistry, catalytic properties and pharmacological application [22,23,24,25,26]. Here we focus on patellamides, which were discovered in 1981 by researchers in search of bioactive molecules for medical applications. Collected specimens of the didemnid ascidian *Lissoclinum patella* from Palau were homogenized, the organic molecules extracted, separated and the activity of the compounds evaluated [27]. The compounds showed interesting pharmacological properties (see below) and were named after its putative producer. While large amounts of patellamides are found in all parts of the ascidian [28,29], *Lissoclinum patella* does not appear to possess the genes required for patellamide biosynthesis [30]. Instead, patellamide biosynthesis occurs in the symbiotic cyanobacterium *Prochloron* that colonizes didemnid ascidians [31] but it remains unclear why *Prochloron* produces patellamides and how its expression is regulated in the ascidian-*Prochloron* symbiosis. It is noteworthy that in *Lissoclinum patella*, another bacterial symbiont solely appears to synthesize cytotoxic macrocyclic molecules, the patellazoles [32,33], unlike *Prochloron*, which primarily acts as a photosymbiont. Metabolic profiling and transcriptomic analysis of natural ascidian-*Prochloron* samples show a high constitutive expression of *patA* and *patE*, two genes involved in patellamide biosynthesis during both night and daytime [30]. This indicates that patellamides might play an essential role in the ascidian-*Prochloron* symbiosis. In the following sections, we review the discovery of patellamide compounds along with the basic structural properties of the macrocycles and their metal complexes (specifically copper(II)) and their properties in biologically relevant catalytic reactions.

## 2. Isolation, Synthesis, Molecular and Biological Properties of Patellamides and Their Metal Complexes

### 2.1. Medical Applications and Structures of Metal-Free Patellamides

Many cyclic compounds from marine organisms were found in drug-discovery screening projects [34,35,36,37,38,39,40,41,42,43,44], including patellamides, which were initially discovered by medical researchers looking for marine metabolites with antineoplastic properties [27,45,46,47,48,49]. Patellamides demonstrated cytotoxicity against L1210 murine leukemia cells with an IC_50_ of 2–4 µg mL^−1^, and patellamide A also showed activity against the human ALL cell line CEM with an ID_50_ of 0.028 µg mL^−1^ [27]. Moreover, patellamide D was shown to reverse multidrug resistance in the human leukemic cell line, presumably by competitively binding to p-gp and by blocking transport proteins involved in causing drug resistance [50]. There is continuous interest in naturally occurring cyclic peptides for potential medical applications and while many bioactivity screening studies are performed with extracts from collected ascidian specimens, synthetic derivatives have also been extensively used [26,35,36,40,51,52,53,54,55].

**Figure 2:**
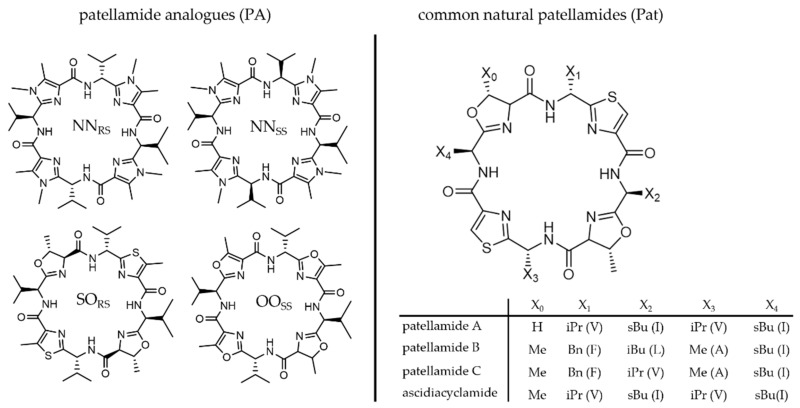
Comparison of some commonly used patellamide analogues (PA, left) and some of the most common natural patellamide derivatives (Pat) with the variable macrocycle side chains X_1_–X_4_ derived from the corresponding amino acid (mentioned as one-letter-codes in brackets). Comprehensive lists of natural and synthetic patellamide derivatives are given elsewhere [23,57].

The first structural proposed structure of an isolated patellamide [27] included directly connected thiazole and oxazoline heterocycles and was later corrected in combination with a proposed synthetic pathway for natural patellamides [56]. Usually, the patellamide heterocycles consist of two aromatic thiazole and two oxazoline rings with a methyl substituent α to the oxygen atom and in *trans* configuration with respect to the adjacent amide. The heterocycles are formed between the heteroatom of a polar amino acid used in the biosynthetic pathway (oxygen in case of serine or threonine, sulfur in case of cysteine) and the peptide bond between this polar amino acid and the non-polar amino acid at its N-terminus. Imidazole heterocycles are not observed due to the lack of an amine analogue of serine in the ribosome [23]. The side chain present between the heterocycles varies in the natural derivatives and depends on the amino acid used in the synthesis, i.e., methyl from alanine, benzyl from phenylalanine, isopropyl from valine or isobutyl derived from isoleucine. It is not clear why there is such a variety of naturally occurring patellamide derivatives and whether these lead to different biological functions. All patellamides identified in ascidian extracts share common structural properties (Figure 2). The natural macrocycles and the synthetic derivatives are cyclic *pseudo*-octapeptides, where four of the peptide bonds are cyclized to form heterocycles, leading to a 24-membered macrocycle with four heterocycles. The relatively rigid backbone contains eight nitrogen atoms, each separated by two carbon atoms. Four of the nitrogen atoms are part of the remaining peptide bonds, while the other four are part of the heterocycles. Members of the patellamide family mostly differ in the pendent side chains at the macrocycle backbone, and the stereochemistry of the four substituents generally is *R,S,R,S*. That is, the amino acid at the N-terminus of the thiazoles is always a *d*-amino acid, while the amino acids at the N-terminus of the oxazoline always are *l*-amino acids. In the biosynthetic pathway, this requires an intentional epimerization of the originally available *l*-amino acid. 

In natural patellamides, two thiazoles and two 2-oxazolines alternate within the cycle. Since the oxazoline results from cyclization of the heteroatom of either a serine or threonine amino acid side chain with the adjacent peptide bond of the *l*-amino acid at its N-terminus, either an (*R*)-5-methyl-4,5-dihydrooxazoline or 4,5-dihydrooxazoline is produced. Oxazoline without a 5-methyl group is only found in patellamide A, while all other known natural derivatives have a 5-methyl group (see Figure 2). Note that this methyl group has (*R*)-configuration [58], and the naturally occurring threonine would lead to the inverse stereochemistry [59]. Therefore, enzymes inverting the stereochemistry of the methyl group are involved in the biosynthesis [58]. This might suggest that the methyl groups at the heterocycles provide an important function. Interestingly, in patellamide A, only one of these methyl groups exists as the other oxazoline is derived from serine. To obtain the thiazoles from the cyclization product thiazoline, another distinct enzyme is needed to oxidize (or cyclo-dehydrogenize) the heterocycle [58], suggesting that the presence of two aromatic thiazoles and two non-oxidized oxazolines are required for the biological function.

**Figure 3:**
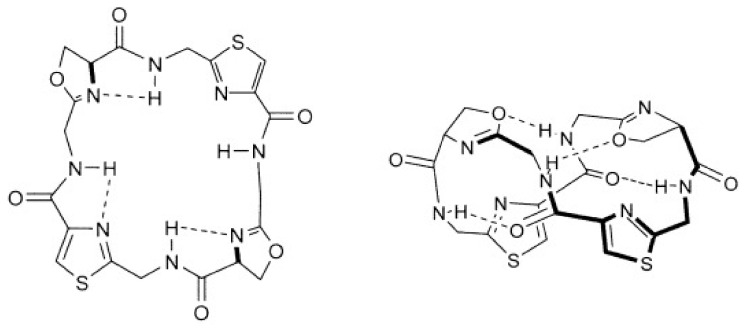
Comparison of the saddle/square shaped conformation (left) and the figure-of-eight conformation of patellamides (reproduced with permission from [60]).

Structural properties of patellamides in solution and in the solid state have been investigated to better understand the behavior of the macrocycles and their metal complexes and to potentially derive biological properties and functions [48,56,61,62,63,64,65,66,67,68,69,70,71,72]. Due to the four heterocycles, the NH-groups of the four peptide units always point towards the inside of the cycle, while the side chains are oriented in pseudo-axial positions on the outside, where the specific orientation depends on the stereochemistry of the amino acid (see structural discussion of the copper(II) complexes below). A series of X-ray crystallographic and NMR spectroscopic studies, combined with computational modeling, has shown that the macrocycles in patellamides assume either a “saddle“ or “figure-of-eight” shaped conformation both in solution and in solid phase (see Figure 3). Which of these geometries is more stable depends on the heterocycles as well as the side chains of the macrocycle [48,63,65,66,67,68,73,74,75]. The corresponding conformational equilibria are of particular importance for the binding of metal ions (see below), and the coordination of the first of two copper(II) ions to the patellamide generally leads to a structural change from the “figure-of-eight” shape towards the “saddle” shape, and this is ideal for the cooperative binding of a second copper(II) ion [76,77]. Notably, this peculiar behavior seems to be specific for copper(II) and causes patellamides to have a relatively high binding affinity and therefore selectivity for copper(II). While the absolute value of copper(II) complex stabilities are not high compared to molecules specifically optimized for strong binding to copper(II), patellamides do occur in an environment that is naturally enriched in copper(II) (didemnid ascidians can contain a 10^4^-fold higher copper(II) concentration than the surrounding seawater [78]). Taken together, this suggests that copper(II) complexation might be connected to the biological function of the patellamides.

### 2.2. Patellamide Syntheses 

**Figure 4:**
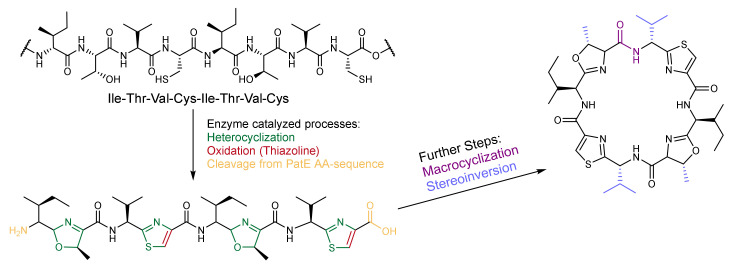
The biosynthetic approach for the synthesis of patellamides. The genes encoding the initial precursor sequence as well as some of the enzymes required for the various steps have been identified and successfully used to biosynthetically produce patellamides in vitro or in *E. coli* [29,58,79,80,81,82,83,84,85]. While many steps during the biosynthesis in *Prochloron* spp. have been studied, processes like the stereoinversion are not fully understood yet.

Initially, patellamides were isolated from biological material (see above). In parallel to conventional synthetic approaches described below, various biosynthetic and hybrid methods to produce patellamides were developed. The gene encoding for patG was introduced and expressed within *Escherichia coli* to study the natural synthesis pathway of patellamide A [29]. The findings from these experiments allowed to develop approaches to obtain a wide range of different marine molecules within cultures of *Escherichia coli* [79] (see Figure 4 for a general Scheme of the biosynthesis). In addition, synthetic peptide coupling steps in solution and solid phase were combined with biosynthetic approaches in an attempt to obtain the natural derivatives [58,80,81,82,83,84,85].

**Figure 5:**
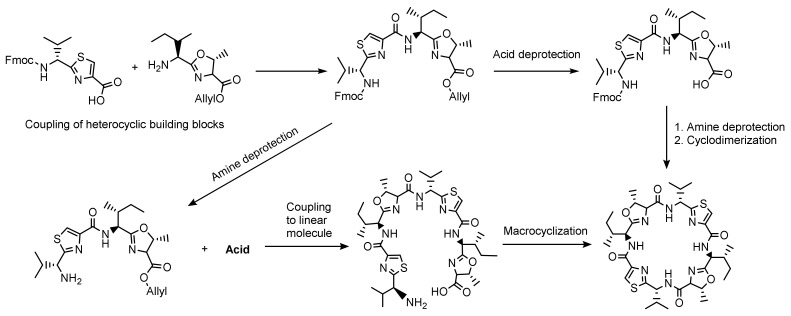
Example of a convergent synthetic approach towards the synthesis of ascidiacyclamide starting from building blocks formed beforehand by coupling and cyclization of two amino acids, in this example valine with *l*-cysteine and *l*-isoleucine with *l-allo*-threonine [57]. All known total synthetic approaches require coupling, cyclization steps for the heterocycles, a cyclization step for the macrocycle, and the application of protective groups.

After tedious synthetic procedures leading to natural product analogues [59,86], and first attempts to prepare patellamide-derived ligands for copper(II) coordination chemistry [87], a big step forward was an elegant and widely variable synthesis of patellamide-like macrocycles developed by Haberhauer and coworkers [72,88]. This enabled the preparation of non-natural patellamide analogues with various heterocycles (including imidazole), pendent groups at their macrocycle backbone, as well as different stereochemical properties. The use of synthetic patellamide analogues for medical testing, copper(II) coordination chemistry and biological studies (see below) has the advantages that the derivatives are relatively easy to obtain and do not require destructive sampling of biological material and that their properties can be tested and optimized as a function of a wide variation of structural and electronic properties. Previous studies have shown that even small structural differences between patellamide analogues can lead to changes in their binding properties [78,87,89]. Different model compounds may therefore exhibit different functional properties and may also differ from the naturally occurring patellamide macrocycles. Haberhauer and Rominger [88] developed a relatively fast, cheap and simple synthetic pathway for patellamide analogues with methylated thiazole and imidazole heterocycles and simple side chains with variable stereochemistry. Some of the most important analogues prepared using this method include PANN_SS_, PANN_RS_, PAOO_SS_ and PASO_RS_ (Figure 2). In many of these analogues, all four side chains are isopropyl groups derived from valine, compared to the naturally occurring isobutyl (isoleucine), benzyl (phenylalanine) or methyl (alanine) groups, and in the simplest analogues the stereochemistry is the same for all four groups. Due to their accessibility, these compounds were also used to assess the catalytic properties of patellamide–copper(II) complexes (see below). The original synthetic approach [90] was updated in 2008 [59] together with a new synthesis route for patellamide A, using Fmoc as protecting group and new cyclization methods for the heterocycle formation such as an approach using Burgess-Reagent [91]. Recently, these previous preparative methods were combined and further adapted to allow for a flexible, convergent, Fmoc-based synthesis of patellamide derivatives, involving the coupling of heterocyclic building blocks to basically obtain any desired patellamide derivative (see Figure 5) [57]. 

### 2.3. Structural Properties of Patellamide Complexes

The shape of the patellamide macrocycle with eight nitrogen donors, the observation of a 10^4^ fold increase of copper(II) concentration in ascidians (see above), and some early observations of metal ion coordination to patellamide derivatives has sparked the interest of coordination and bioinorganic chemists in naturally occurring patellamides. This involves unravelling their synthesis and in particular the solution and structural chemistry of patellamide complexes, primarily with copper(II), as well as in the reactivity of these patellamide complexes [23,25,46,60,77,86,87,92,93,94].

Patellamide derivatives are larger, have more donor groups and are more flexible than other biologically relevant macrocycles such as porphyrin derivatives, and it therefore is not unexpected that they are well-suited to bind two metal ions [23]. In fact, it has been shown that there is cooperative binding of two copper(II) ions to patellamide derivatives [23,60,76], and even the smaller macrocycles (westiellamides, pseudo-hexapeptides, 18- vs. 24-membered rings) have been shown to form dinuclear copper(II) species [95,96]. Due to the high concentrations of copper(II) ions in the ascidians compared to the sea water, the published patellamide coordination chemistry is mainly focused on copper(II), with few studies also involving zinc(II) [77,97] and very few with other metal ions [23]. We note that there are only relatively few X-ray crystal structures of complexes with patellamide derivatives. However, the solution structures are generally more important and revealing for their reactivity and possible biological function. For dinuclear copper(II) complexes, a method based on EPR spectroscopy combined with spectral simulations and structural modeling has been developed [98] and used extensively to determine the structural properties of copper-patellamide complexes [23,60,76,87].

**Figure 6:**
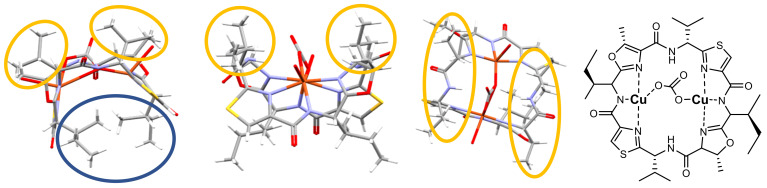
Experimentally determined 3-dimensional structure of the patellamide-dicopper(II) complex of ascidiacyclamide with a bridging carbonate shown in three different orientations with the molecular structure of the complex shown on the right (the water molecules bound to the copper are omitted for clarity) [99]. The perspectives indicate that the two *sec*-butyl side chains derived from *R*-isoleucine (marked in blue) block the reactive center from one side, while the two isopropyl side chains originating from *S*-valine together with the methyl groups on the oxazoline heterocycles form a well-defined pocket at the dicopper(II) site.

Structural and stereochemical features of copper(II) coordination to patellamide macrocycles are visualized in Figure 6 with the carbonato-bridged dicopper(II) complex of ascidiacyclamide, where each copper(II) center is coordinated to three of the nitrogen atoms from the macrocycle, two heterocycles and one amide. In dicopper(II) complexes of patellamides two amide groups are deprotonated, leading to a dianionic macrocyclic ligand coordinated to the two copper(II) cations. In the resulting saddle-shaped structure (Figure 3 and Figure 6), the *sec*-butyl side chains derived from *l*-isoleucine (blue circle) might hamper coordination of molecules (e.g., substrates in catalytic transformations, see below) to the two copper(II) ions from the bottom, while the smaller *iso*-propyl substituents derived from *d*-valine (orange circles) leave an open channel at the top that might help to control the pathway for a possible substrate interaction. Interestingly, the two *iso*-propyl side chains of ascidiacylamide are in line with the methyl groups from the oxazoline heterocycles (Figure 6, second from right) and therefore build a well-defined pocket for substrate attack at the dinuclear metal site (see also section below on catalysis). 

Comparative studies of the copper(II) complex stabilities with patellamide A and patellamide C indicate that the metal ion affinities of these two cyclic peptides are rather different, although the two ligands differ only in the structure of the pendant groups at the backbone and the number of methyl groups at the oxazoline heterocycles (see Figure 2). The published complex stability constants of copper(II) for the two derivatives are 2.0 × 10^4^ for patellamide A and 6.8 × 10^4^ for patellamide C [89]. This difference suggests that the nature of the side chains and their stereochemistry at the backbone and the heterocycles are of some importance for the molecular properties of patellamide complexes and that this might also be relevant for their natural role. 

### 2.4. Catalytic Properties of Patellamide Complexes

Carbonato-bridged dicopper(II) complexes were among the first copper(II)-patellamide complexes reported, including a crystal structure based on the natural ascidiacyclamide species [99]. Importantly, a study of the solution equilibria of patellamide derivatives with copper(II) based on optical (UV-VIS-NIR, CD) and EPR spectroscopy indicated that the various equilibria are shifted in presence of air, and this could be traced back to the hydrolysis of CO_2_ and coordination of carbonate, i.e., carboanhydrase activity [60]. Indeed, a thorough mechanistic study indicated that the dicopper(II) complexes of patellamide derivatives are very efficient carboanhydrase mimics, in fact, they are the most efficient synthetic carboanhydrase models known to date and only about two orders of magnitude slower than the human carbonic anhydrase [23,100]. The assumed mechanism is based on isotope labeling experiments and stopped-flow kinetics, and has also been supported by quantum-chemical calculations [101]. This is an interesting observation because all corresponding carboanhydrase enzymes are mononuclear zinc(II) enzymes. Whether such efficient CO_2_ hydrolysis is of importance in the ascidian-*Prochloron* symbiosis remains to be investigated in further detail. Experiments with an imidazole-based patellamide analogue, decorated with a fluorescent dye in living *Prochloron* cells, indicates that copper(II) is coordinated to these ligands in vivo [102]. Copper(II) complexes of patellamide analogues have also revealed a range of other catalytic abilities, including those of phosphomono- and -diesterase [97,103], as well as lactamase and β-glycosidase [104].

Most kinetic experiments related to the catalytic efficiencies of patellamide complexes were studied by time-dependent optical spectroscopy (UV-VIS-NIR, CD, including stopped-flow techniques) in aqueous solution under standardized conditions, where optical changes of substrates or additives were monitored as a function of time. For in vivo experiments, biocompatible assays (e.g., based on fluorescence) need to be developed. Such targeted in situ measurements might also provide detailed insights into the effects of the dynamic host microenvironment (discussed below) on the catalytic behavior of different patellamides. 

While all catalytic reactions described above might have biological importance, we note that only the carboanhydrase activity has so far been shown to be very high. However, most of these reactions have not been studied in detail as a function of the type and stereochemistry of the macrocycle substituents, and most of the reactions studied used the artificial imidazole derivatives. Therefore, one needs to be cautious in terms of assigning biological relevance to these observations. An interesting aspect is that the various catalytic reactions studied so far operate in different optimal pH ranges. This is relevant with respect to the various pH values observed in *L. patella* at different times of the day and in different parts of the animal (see below). 

## 3. The Symbiotic Backdrop—A Highly Dynamic Place of Patellamide Production

The structural and optical properties of the ascidian tunic modulate both the internal and external microenvironment of the didemnid ascidian host and its associated microorganisms (Figure 1). Filter-feeding ascidian zooids can move water via ciliary activity on their pharynx into the joint cloacal cavity of the colony that is typically connected to the surrounding seawater via one or few exhalent openings. Didemnid ascidians like *L. patella* are closely associated with their underlying substratum (e.g., old coral skeleton) and are embedded in the benthic boundary layer. They thus experience much lower flow velocities as compared to the overlying, turbulent seawater and exhibit low pumping rates that processes only a minor amount of the water flowing over the colony [105]. Besides the inhalant and exhalant openings, the ascidian colony is surrounded by a diffusive boundary layer, where mass transfer by diffusion prevails over advective transport leading to formation of steep concentration gradients of, e.g., O_2_ between the seawater and the ascidian tunic, which change dynamically with incident irradiance [106]. The cohesive structure of the tunic and the intricate system of narrow cloacae in *L. patella* and similar didemnid ascidians also represent a diffusion barrier for solutes, which leads to dramatic changes in the internal chemical microenvironment between light and darkness in the ascidians, especially in the zone colonized by *Prochloron* [106,107]. Furthermore, the optical properties of the tunic modulate the internal light field and thus the light microclimate of *Prochloron* and other phototrophs associated with didemnid ascidians, due to scattering of light in the tunic and embedded spicules, as well the presence of UV absorbing compounds in the tunic [108,109]. This leads to steep light gradients across the ascidian colony [106,107]. Microbial life on and within didemnid ascidians thus takes place in a dynamic microenvironment characterized by steep gradients of light and chemical parameters that are modulated by both metabolic processes and mass transfer impedance. 

### 3.1. Microenvironments and Biological Dynamics of the Ascidian-Prochloron Symbiosis

The ascidian holobiont contains a large diversity of microorganisms, often separated by only a few millimeters of ascidian tissue. *L. patella*, for example, harbors distinct microbial biofilms on its surface, cloacal cavity and underside, and each of these microniches is characterized by its own unique set of physico-chemical parameters, potentially influencing the production and functioning of patellamides [106,110]. Despite this knowledge, most studies on ascidians have relied on bulk sampling and thereby integrated important details on the structure and function of microbial community ensembles into average values. To provide resolution to our current understanding of the biological dynamics on ascidians, we give a detailed description of ascidian microniches, explore the diversity and functioning of resident microbial communities and, finally, hypothesize on their joint influence on patellamide production and functioning.

#### 3.1.1. The Outer Surface and Tunic of Ascidians

The upper surface microenvironment of didemnid ascidians is characterized by strong light scattering in the tunic, which can lead to locally increased irradiances of ~140–170% of incident light [106,107]. Oxygen levels on the surface dynamically change in response to ambient light levels, which can stimulate photosynthesis in surface-residing phototrophs (or in the cloacal cavity below). However, oxygen microsensor measurements also revealed decreasing O_2_ levels in the ascidian subsurface (~1 mm into the animal), likely an effect of animal respiration [110]. pH conditions on the surface do not change as acutely as in the cloacal cavity or the underside of ascidians, where increasing levels of incident photon irradiance lead to the buildup of steep pH gradients. Imaging of the surface of *L. patella* revealed the presence of filamentous cyanobacteria, often present in conspicuous ‘tufts’ lining the water intake of the zooid animal [110] and resembling previously described morphotypes from the genus *Planktothricoides* [111]. Parallel DNA sequencing of *L. patella* surface microbiomes revealed it to be somewhat diverse (mean Shannon diversity = 3.3) and dominated by the phyla cyanobacteria (e.g., Planktothricoides), proteobacteria (e.g., Kiloniellaceae) and bacteroidetes (e.g., Flammeovirgaceae) [110]. Alpha- and beta-proteobacteria were also frequent community members on the surface of *Didemnum galacteum* and *Cystodytes* sp. [112] and, surprisingly, so were *Prochloron*-like cells, which supports earlier descriptions of facultative associations between ascidians and *Prochloron* cells [3,113]. Further support comes from recent findings where *Prochloron* was present on the surface of crustose didemnid ascidians and exhibited a much larger phylogenetic diversity compared to obligate *Prochloron* cells [4]. Whether these surface-associated *Prochloron* cells produce patellamides or whether they are more amenable to cultivation remains to be investigated and presents an exciting avenue, as it might simplify the *in vitro* production of patellamides. In summary, current data indicates that the surface of didemnid ascidians is characterized by (i) moderate variation in O_2_ and pH between light and darkness, (ii) moderate microbial diversity and the occasional dominance by surface-associated *Prochloron* cells, (iii) an essentially unknown potential for patellamide production.

#### 3.1.2. The Cloacal Cavity of Ascidians

**Figure 7:**
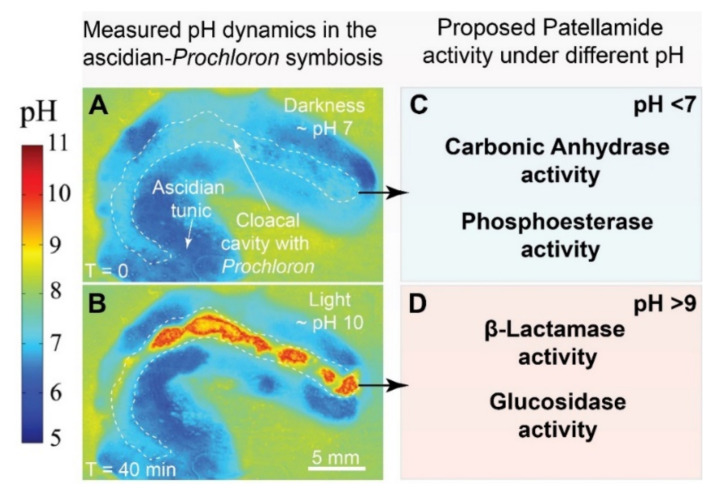
Suggested effects of pH microenvironments in *Lissoclinum patella* on the functioning of patellamide-copper complexes. (**A**) Pseudocolor images of measured pH distributions in the ascidian-*Prochloron* symbiosis in darkness (= T_0_ minutes). Note the low pH (~6–7) in the cloacal cavity that contains *Prochloron* cells. (**B**) Measured pH distributions in the ascidian-*Prochloron* symbiosis after 40 minutes of moderate irradiation (250 µmol photons m^−2^ s^−1^). Note the elevated pH (~9–10) in the cloacal cavity that contains photosynthetic *Prochloron* cells. Figure 7A,B are partially redrawn from Figure 7 in [106]. (**C**) The catalytic activity spectrum of patellamide-copper complexes at pH < 7. (**D**) The catalytic activity spectrum of patellamide-copper complexes at pH > 9.

The inner cloacal cavity and upper tunic of many didemnid ascidians is conspicuously lined with deep green *Prochloron* symbionts (e.g., [10,11]). Compared to the surface of ascidians, light levels in the cloacal cavity are approximately 1/10–1/100 of the incident downwelling irradiance [106], mostly due to the strong visible light absorption by *Prochloron* itself, and the depletion of harmful UV radiation due to the presence of UV-screening compounds, i.e., mycosporine-like amino acids, in the upper tunic [109]. Upon exposure to high levels of incident photon irradiance (e.g., 1350 µmol photons m^−2^ s^−1^), oxygen levels in the cloacal cavity rapidly reach supersaturation (150–400% of air saturation), while darkness induces full anoxia (0% of air saturation) within a few minutes [106,107]. The photosynthetic activity of *Prochloron* also influences the local pH microenvironment in the cloacal cavity. Moderate irradiance levels, i.e., 250 µmol photons m^−2^ s^−1^, induced an upshift from ~pH 7 to pH 10 within 20 min in the *Prochloron*-containing cloacal cavity of *L. patella* (Figure 7A,B) [106]. The cloacal cavity is thus characterized by rapidly changing levels of light, O_2_ and pH, and represents a dynamic chemical microniche, which ostensibly influences the biosynthesis and catalytic activity of patellamides but also selects for a highly specialized microbiome. While most molecular surveys have targeted the tunic of whole animals, some have investigated the cloacal cavity (in)directly, e.g., by gently squeezing the animal host to release microbial cells. This revealed a relatively low-diversity (mean Shannon diversity = 1.87) and a dominance of cyanobacteria, specifically *Prochloron*, and proteobacteria within the cloacal cavity of animals [110,114]. A geographic survey of *Prochloron* cells (pressed out of cloacal cavities of various ascidian species) revealed surprisingly little differences between the genomes of distant *Prochloron* isolates, albeit with some degree of diversification among their secondary metabolite pathways [115,116], which corroborates that some, but not all, didemnid ascidians contain patellamides [31]. The analysis of entire didemnid ascidians, i.e., not limited to only their cloacal cavity, revealed a high degree of bacterial host specificity [117,118] and the existence of a *Prochloron*-dominated core microbiome for *L. patella* alongside a distinct production of secondary metabolites in the same species [119]. Follow-up work by Lopez-Guzman [120] confirmed this host specificity but also revealed a cryptic diversity of *Prochloron* in the cloacal cavity of Japanese ascidians, possibly related to different modes of symbiont acquisition (reviewed in detail in [3]). Notably, the same study also found that some didemnid ascidians did not contain *Prochloron* cells at all and instead were dominaned by other cyanobacterial symbionts. Taken together, this suggests that the photosymbiotic association between *Prochloron* and didemnid hosts is the rule, rather than the exception, among these ascidians. In addition, the cloacal cavity of many, but not all, didemnid ascidians appears to be dominated by *Prochloron* and other cyanobacteria, which actively engage in the production of diverse secondary metabolites, including patellamides. However, the effect of changing physico-chemical microenvironments on *Prochloron* metabolism and patellamide functioning remains essentially unknown.

In an attempt to better understand this interplay, transcriptomics and metabolomics were recently used on samples collected from the cloacal cavity of *L. patella* during midday and midnight [30]. This revealed that, during midday, *Prochloron* dynamically upregulates photosynthesis-related genes (e.g., *psbA*) as well as genes involved in UV-protection and that the ascidian metabolome becomes dominated by amino acids (e.g., Asp, His, Gly) and sugars (e.g., ribose, maltose, fucose). During midnight, *Prochloron* upregulates genes involved in the formation of RuBisCO (e.g., *rbcL*, *rbcS*, *rbcX*) and carbonic-anhydrase, and the cloacal metabolome becomes enriched in host-derived neurotransmitters (e.g., GABA) and sugar acids (e.g., galactonic acid). Diel changes in microenvironments did not change *Prochlorons*’ expression of genes involved in nitrogen utilization (e.g., *glsF*, *ureC*) but instead the abundance of citrulline, a chemical intermediate in the urea cycle, which was measured at higher concentrations during midday. Together, this suggests that *Prochloron* continuously ‘frontloads’ nitrogen-utilizing proteins to be prepared for host-derived urea during light conditions. These findings are in line with the expectation that the photosynthesis, carbon fixation and nitrogen utilization pathways of *Prochloron* are responding to microenvironmental changes that occur during a diel cycle. Surprisingly, the expression of genes coding for parts of patellamide precursors (i.e., *patA*, *patE*) did not change during midday or midnight and these genes were expressed at levels similar to photosynthesis housekeeping genes. The continuous and thus costly expression of patellamides highlights their important role for the survival and fitness of *Prochloron*. We hypothesize that the multifaceted function of patellamides under specific microenvironmental conditions, e.g., different catalytic functionality over the span of observed pH dynamics in hospite, could imbue *Prochloron* with a ‘chemically-mediated’ advantage over its microbial competitors (Figure 6). To summarize, current research indicates that the *Prochloron*-containing cloacal cavity is characterized by (i) highly fluctuating levels of O_2_ (ranging from anoxia to hyperoxia) and pH (ranging from pH ~6 to pH ~10) under dark and light conditions, (ii) a dominance by *Prochloron* and other cyanobacteria, (iii) a transcriptome and metabolome that responds to diel changes in light and associated microenvironmental changes, and (iv) a continuous and very high expression of selected genes coding for patellamides.

#### 3.1.3. The Underside of Ascidians

Ascidian larvae seek out hard substrates, which provides the animal with a structured topography for subsequent attachment and growth. The underside of ascidians thus represents an important abiotic-biotic interface, which is characterized by its own unique microenvironment and resident microorganisms. Naturally, light levels on the underside of an ascidian are much lower compared to its surface or cloacal cavity and, in the case of didemnid ascidians, comprise only approximately 1/100th of the incident downwelling irradiance compared to the surface [106,110,121]. However, this attenuation is much less dramatic for wavelengths in the red part of the spectrum (>700 nm), which propagate quite effectively through the tunic and cloacal cavity (which contains chlorophyll *a*/*b* produced by *Prochloron*) and reach the underside [121]. Probing the underside of ascidians with amperometric microsensors is difficult as they are fragile and prone to breaking, but measurements with 2D planar optodes demonstrated light-dependent shifts in O_2_ concentration and pH in biofilms on the underside of *L. patella* [106]. Upon illumination with moderate photon irradiances (250 µmol photons m^−2^ s^−1^), oxygen levels in microbial biofilms on the underside of *L. patella* increased to ~ 65% air saturation, while darkening induced hypoxia (~10–20% air saturation). Compared to the other microenvironments found in ascidians, the underside is thus exposed to low photon irradiances, predominated by red wavelengths and experiences only moderate changes in pH and O_2_. Amplicon sequencing of DNA extracted from the underside of *L. patella* revealed this location to harbor a diverse microbiome (mean Shannon diversity = 6.93) and to be dominated by representatives from the phyla cyanobacteria and proteobacteria [110]. *Prochloron* appears absent from the underside of *L. patella* [110], which instead is frequently inhabited by *Acaryochloris marina*, a cyanobacterium that benefits from the enrichment in near infrared wavelengths by using chlorophyll *d* for oxygenic photosynthesis [30,121,122,123,124]. Further molecular investigation of *A. marina* in association with the underside of *L. patella* demonstrated that this cyanobacterium modulates the expression of photosynthesis-related genes during noon and midnight, while other microorganisms express genes involved in sulfate metabolism (*cysD*), the production of secondary metabolites (*TOMM C/D*), and pathogenicity (*Invasin*) [30]. To our knowledge, no other studies have specifically investigated the underside of ascidians and our limited data indicate that this habitat is characterized by (i) moderate changes in O_2_ and pH, (ii) low irradiance with a predominance of near infrared light, (iii) a dominance of cyano- and proteobacteria but an absence of *Prochloron*, and (iv) the production of unknown secondary metabolites. 

### 3.2. Possible Functions of Patellamides in the Ascidian-Prochloron Symbiosis

*Prochloron* provides its host with photosynthesis products [19] and this might be its primary role. Therefore, it remains possible that patellamides might ultimately not provide any functional advantage to the host. Keeping this in mind, there are two important facts: (i) the copper concentration in ascidians is high (10^4^ times higher than in the sea water and relatively high compared to other biologically relevant metal ions) [23,25,78,93,125], and (ii) the patellamide macrocycles are abundant in ascidians (see above) [28,29]. Copper thus appears important for the ascidian-*Prochloron* symbiosis, and it is likely that patellamide macrocycles play a major role for the activity revealed by copper. There is increasing evidence that copper(II) is coordinated to patellamides in ascidians and *Prochloron*. The most compelling confirmation comes from (i) photophysical experiments involving a photoactive patellamide analogue inside living *Prochloron* cells [100], and (ii) recent support from preliminary synchrotron experiments involving model compounds and frozen biological material [126].

While there is no direct proof that complexation of copper(II) by the patellamides is vital for the ascidians, the evidence is quite compelling. In terms of the biological role for the copper(II)-patellamide complexes, there are various interesting and sensible ideas, but these are without experimental evidence, i.e., these are educated guesses at most. The main proposed functions encompass (i) metal ion sequestration and/or transport, (ii) protection from predators, and (iii) catalysis and/or transport of substrates. 

#### 3.2.1. Metal Ion Sequestration and Transport

It is an interesting question why copper is important for *Prochloron* or the ascidians. The first concern to take care of is how copper(II) is selectively transported into the ascidian-*Prochloron* system. It is known that patellamide derivatives have moderate complex stability constants with copper(II) but, more importantly, that copper(II) stabilities are generally 1 to 2 orders of magnitude larger than for zinc(II) and significantly larger than for calcium(II) [23,25]. While the data basis is not large and not easily comparable, based on copper(II)-patellamide complementarity, preorganization, cooperativity, and electronic (e.g., the Irving-Williams series) and thermodynamic expectations (chelate and macrocycle effects), the observed copper(II) selectivity supports a possible role for the patellamides in the accumulation as well as transport of copper(II) in ascidians and *Prochloron*. However, it remains an open question why so many structurally different macrocycles are produced.

#### 3.2.2. Protection

Many of the cyclic peptides found in marine organisms have been assumed to have a defensive function for the host and its microbiome [127,128]. It is therefore not surprising that one of the first ecological roles assigned to patellamides was that of a defense molecule [129]. However, toxicological studies could neither identify adverse effects of patellamides on potential predators and microorganisms nor indicate a change in palatability [129,130]. However, these studies primarily refer to the metal-free peptides and not copper(II)-patellamide complexes, which will likely have different toxic properties.

#### 3.2.3. Catalysis and Transport of Substrates

Copper is essential in biosystems and many copper-proteins are known to be involved in electron transfer and oxygen activation processes. Based on the efficient copper(II)-patellamide-catalyzed hydrolyses discussed above, it is possible that copper-patellamides are involved in the transformation of organic substrates, in electron transfer or the transport of important small molecules such as O_2_ and CO_2_ and their derivatives. The large variety of patellamide macrocycles in the ascidians might lead to activity for one or several reactions depending on the patellamide derivative, pH, and redox conditions. However, this is pure speculation, and none of the proposed functions has been shown to be relevant in vivo. Generally, high concentrations of metal ion and the ligand are not required for enzymatic catalysis. That is, the high concentration of copper(II) in comparison to zinc(II) does not mean that catalytic hydrolyses (e.g., carboanhydrase and phosphatase), usually performed by zinc enzymes, are based on copper enzymes in ascidians and *Prochloron*. It would be interesting if a redox-active metal ion, such as copper, is involved in enzymatic hydrolysis (carboanhydrase, phosphatase, β-lactamase, glycosidase, see Figure 7). While there is some precedence for this in the iron(III)-containing purple acid phosphatases [131], such function of patellamides remains speculative due to lack of experimental data.

The proposed role of patellamides in oxygen activation [93] is primarily based on the structure of their dicopper(II) complexes (see Figure 6 for a visualization of a similar molecule). Structurally, the formation of well-known dicopper-peroxo and dioxido complexes is possible, but the donor set provided by the patellamides suggests that the required copper(I) precursor is likely unstable, which is supported by preliminary electrochemical experiments [76]. While oxygen activation and electron transfer might be possible with patellamides as prosthetic groups in proteins such as with the heme enzymes, no experimental evidence for this exists.

The observed carboanhydrase activity [91,99,101] is of particular interest because the catalytic activity of the dicopper(II)-patellamide complexes studied in vitro is close to that of zinc(II) enzymes. Their in vitro phosphatase, β-lactamase, and glycosidase activities are much lower, but depending on the environment in the ascidians and the structure of the patellamide derivative, this might be different in vivo. An interesting observation is that dicopper(II) complexes of patellamide macrocycles are complementary for carbonate binding and that this is part of the carboanhydrase catalytic cycle [88,91,98,99,101]. Therefore, even if the copper-patellamide complexes would not function as carboanhydrases (they might also be a backup system for the conventional zinc(II) carboanhydrases), they might be involved in carbonate transport from the ascidian to the photosynthetic symbiont *Prochloron*.

## 4. Summary and the Future of Patellamide Research

Patellamides are bioactive cyclic peptides that have been intensively studied with respect to their structural dynamics, pharmacological applications, metal ion (specifically copper(II)) solution chemistry, and the reactivity of the corresponding copper(II)-patellamide complexes. In nature, the cyclic octapeptides are exclusively produced by *Prochloron didemni*, a unicellular cyanobacterium that engages in photosymbiosis with marine didemnid ascidians. Microbial life on and within didemnid ascidians takes place in distinct dynamic microenvironments that modulate metabolic processes of resident microorganisms. As *Prochloron* cannot be reliably cultivated, the in vitro production of patellamides, primarily by complex organic synthesis of the natural products patellamide analogues, is an important basis for a thorough evaluation of the properties of the cyclic peptides. Extensive in vitro studies of these analogues revealed that the dinuclear copper(II)-patellamide complexes are active catalysts for a range of biologically relevant processes, including CO_2_ and phophoester hydrolysis and glycosyl transfer. As didemnid ascidians are naturally enriched for trace-metals (especially copper) and characterized by dynamically changing levels of O_2_, pH and light, it is conceivable that naturally occurring patellamides are exposed to high copper(II) concentrations and dynamically changing chemical microenvironments. This interplay might play a fundamental role in the function of patellamides in their natural environment.

To advance our knowledge on the interplay between microenvironments and metal ion based patellamide catalysis, it is important to (i) obtain an accurate quantification of patellamides and their distribution in the ascidian, (ii) improve the ability to quantify various chemical constituents such as copper(II) and, more importantly, copper-patellamide complexes, and (iii) evaluate whether patellamide catalyzed reactions also occur in vivo. An important question, particularly in relation to the latter point is, why there is a large variety of patellamide derivatives with subtle structural differences, and how important the emerging structure-function correlations of the copper-patellamide complexes are for the ascidian-*Prochloron* system. Various methods including chemical imaging (e.g., imaging mass-spectrometry and spatial metabolomics) [132], fluorescence-based assays [102], microfluidic technologies [133], and synchrotron-based spectroscopy [126] might provide the ability to assess the importance of the behavior of the copper(II)-patellamide complexes under in vivo like conditions. Such efforts could provide answers to long held questions on the natural function of patellamides and patellamide-copper(II) complexes as well as the problems to cultivate *Prochloron*. 

Understanding these key questions could have larger implications for research on natural products derived from marine organisms. Complex macrocyclic compounds found in marine organisms are, in absence of other evidence, often assumed to have a defensive function. Metal ion binding to the macrocycles and other metabolites has not been considered and studied in enough detail so far. We assume that a thorough understanding of the solution equilibria of macrocycles such as the patellamides in presence of the highly abundant metal ions and the properties of the ensuing adducts, specifically their reactivities, may help to uncover their biological role and lead to novel approaches in the development of synthetic enzymes.

## References

[1] Lewin R.A., Cheng L. (1989). Prochloron: A Microbial Enigma.

[2] Cox G. (1986). Comparison of *Prochloron* from different hosts. I. Structural and ultrastructural characteristics. New Phytol..

[3] Hirose E. (2015). Ascidian photosymbiosis: Diversity of cyanobacterial transmission during embryogenesis. Genesis.

[4] Nielsen D.A., Pernice M., Schliep M., Sablok G., Jeffries T.C., Kühl M., Wangpraseurt D., Ralph P.J., Larkum A.W.D. (2015). Microenvironment and phylogenetic diversity of *Prochloron* inhabiting the surface of crustose didemnid ascidians. Environ. Microbiol..

[5] Maurice C. Etude Monographique d’Une Espece d’Ascidies Composee; Liege, Belgium, 1888; Volume 8. https://books.google.com.hk/books?id=1qoMAQAAIAAJ&ots=wXc2zsas1U&dq=Etude%20Monographique%20d%E2%80%99une%20Espece%20d%E2%80%99Ascidies%20Composee&lr&hl=zh-CN&pg=PA1#v=onepage&q&f=false.

[6] Smith H.G. (1935). LXI.—On the presence of algæ in certain Ascidiacea. Ann. Mag. Nat. Hist..

[7] Tokioka T. (1942). Ascidians found on the mangrove trees in Lwayama Bay, Palau. Palau Trop. Biol. Station Stud..

[8] Lewin R.A. (1977). *Prochloron*, type genus of the Prochlorophyta. Phycologia.

[9] Lewin R.A., Cheng L. (1975). Associations of microscopic algae with didemnid ascidians. Phycologia.

[10] Newcomb E.H., Pugh T.D. (1975). Blue-green algae associated with ascidians of the Great Barrier Reef. Nature.

[11] Lewin R.A., Withers N.W. (1975). Extraordinary pigment composition of a prokaryotic alga. Nature.

[12] Lewin R.A. (1984). *Prochloron*—A status report. Phycologia.

[13] Turner S., Bhattacharya D. (1997). Molecular systematics of oxygenic photosynthetic bacteria. Origins of Algae and Their Plastids.

[14] Roche J.L., van der Staay G.W.M., Partensky F., Ducret A., Aebersold R., Li R., Golden S.S., Hiller R.G., Wrench P.M., Larkum A.W.D. (1996). Independent evolution of the prochlorophyte and green plant chlorophyll *a*/*b* light-harvesting proteins. Proc. Natl. Acad. Sci. USA.

[15] Rumengan I.F.M., Kubelaborbir T.M., Tallei T.E. (2020). Data on the cultivation of *Prochloron* sp. at different salinity levels. Data Brief.

[16] Rumengan I.F.M., Roring V.I.Y., Haedar J.R., Siby M.S., Luntungan A.H., Kolondam B.J., Uria A.R., Wakimoto T. (2021). Ascidian-associated photosymbionts from Manado, Indonesia: Secondary metabolites, bioactivity simulation, and biosynthetic insight. Symbiosis.

[17] Hirose E., Nozawa Y. (2020). Latitudinal difference in the species richness of photosymbiotic ascidians along the east coast of Taiwan. Zool. Stud..

[18] Olson R.R. (1986). Light-enhanced growth of the ascidian *Didemnum molle*/*Prochloron* sp. symbiosis. Mar. Biol..

[19] Kremer B.P., Pardy R., Lewin R.A. (1982). Carbon fixation and photosynthates of *Prochloron*, a green alga symbiotic with an ascidian, *Lissoclinum patella*. Phycologia.

[20] Koike I., Yamamuro M., Pollard P. (1993). Carbon and nitrogen budgets of two ascidians and their symbiont, *Prochloron*, in a tropical seagrass meadow. Aust. J. Mar. Freshw. Res..

[21] Koike I., Suzuki T. (1996). Nutritional diversity of symbiotic ascidians in a Fijian seagrass meadow. Ecol. Res..

[22] Schmidt E.W., Donia M.S., McIntosh J.A., Fricke W.F., Ravel J. (2012). Origin and variation of tunicate secondary metabolites. J. Nat. Prod..

[23] Comba P., Dovalil N., Gahan L.R., Hanson G.R., Westphal M. (2014). Cyclic peptide marine metabolites and Cu^II^. Dalton Trans..

[24] Comba P., Eisenschmidt A., Hanson G., Berliner L. (2017). Structures, electronics and reactivity of copper(II) complexes of the cyclic pseudo-peptides of the ascidians. Future Directions in Metalloprotein and Metalloenzyme Research.

[25] Gahan L.R., Cusack R.M. (2018). Metal complexes of synthetic cyclic peptides. Polyhedron.

[26] Jaspars M., De Pascale D., Andersen J.H., Reyes F., Crawford A.D., Ianora A. (2016). The marine biodiscovery pipeline and ocean medicines of tomorrow. J. Mar. Biol. Assoc. UK.

[27] Ireland C.M., Durso A.R., Newman R.A., Hacker M.P. (1982). Antineoplastic cyclic peptides from the marine tunicate *Lissoclinum patella*. J. Org. Chem..

[28] Salomon C.E., Faulkner D.J. (2002). Localization studies of bioactive cyclic peptides in the ascidian *Lissoclinum patella*. J. Nat. Prod..

[29] Schmidt E.W., Donia M.S. (2010). Life in cellulose houses: Symbiotic bacterial biosynthesis of ascidian drugs and drug leads. Curr. Opin. Biotechnol..

[30] Behrendt L., Raina J.-B., Lutz A., Kot W., Albertsen M., Halkjær-Nielsen P., Sørensen S.J., Larkum A.W., Kühl M. (2018). *In situ* metabolomic- and transcriptomic-profiling of the host-associated cyanobacteria *Prochloron* and *Acaryochloris marina*. ISME J..

[31] Schmidt E., Nelson J., Rasko D., Sudek S., Eisen J., Haygood M., Ravel J. (2005). Patellamide A and C biosynthesis by a microcin-like pathway in *Prochloron didemni*, the cyanobacterial symbiont of *Lissoclinum patella*. Proc. Natl. Acad. Sci. USA.

[32] Lopanik N.B. (2014). Chemical defensive symbioses in the marine environment. Funct. Ecol..

[33] Kwan J.C., Donia M.S., Han A.W., Hirose E., Haygood M.G., Schmidt E.W. (2012). Genome streamlining and chemical defense in a coral reef symbiosis. Proc. Natl. Acad. Sci. USA.

[34] Andavan G.S.B., Lemmens-Gruber R. (2010). Cyclodepsipeptides from marine sponges: Natural agents for drug research. Mar. Drugs.

[35] Stonik V., Fedorov S. (2014). Marine low molecular weight natural products as potential cancer preventive compounds. Mar. Drugs.

[36] Tan L.T. (2013). Pharmaceutical agents from filamentous marine cyanobacteria. Drug Discov. Today.

[37] Tan L.T. (2007). Bioactive natural products from marine cyanobacteria for drug discovery. Phytochemistry.

[38] Zhou X., Liu J., Yang B., Lin X., Yang X.-W., Liu Y. (2013). Marine natural products with anti-HIV activities in the last decade. Curr. Med. Chem..

[39] Zhou Y. (2014). The potential biomedical application of cyclopeptides from marine natural products. Curr. Org. Chem..

[40] Camp D., Davis R.A., Evans-Illidge E.A., Quinn R.J. (2012). Guiding principles for natural product drug discovery. Future Med. Chem..

[41] De Vries D.J., Beart P.M. (1995). Fishing for drugs from the sea: Status and strategies. Trends Pharmacol. Sci..

[42] Kiuru P., D’Auria M., Muller C., Tammela P., Vuorela H., Yli-Kauhaluoma J. (2014). Exploring marine resources for bioactive compounds. Planta Med..

[43] Molinski T.F., Dalisay D.S., Lievens S.L., Saludes J.P. (2009). Drug development from marine natural products. Nat. Rev. Drug Discov..

[44] Salvador-Reyes L.A., Luesch H. (2015). Biological targets and mechanisms of action of natural products from marine cyanobacteria. Nat. Prod. Rep..

[45] Degnan B.M., Hawkins C.J., Lavin M.F., McCaffrey E.J., Parry D.L., Van den Brenk A.L., Watters D.J. (1989). New cyclic peptides with cytotoxic activity from the ascidian *Lissoclinum patella*. J. Med. Chem..

[46] Hawkins C.J., Lavin M.F., Marshall K.A., Van den Brenk A.L., Watters D.J. (1990). Structure-activity relationships of the lissoclinamides: Cytotoxic cyclic peptides from the ascidian *Lissoclinum patella*. J. Med. Chem..

[47] McDonald L.A., Ireland C.M. (1992). Patellamide E: A new cyclic peptide from the ascidian *Lissoclinum patella*. J. Nat. Prod..

[48] Schmitz F.J., Ksebati M.B., Chang J.S., Wang J.L., Hossain M.B., Van der Helm D., Engel M.H., Serban A., Silfer J.A. (1989). Cyclic peptides from the ascidian *Lissoclinum patella*: Conformational analysis of patellamide D by X-ray analysis and molecular modeling. J. Org. Chem..

[49] Rashid M.A., Gustafson K.R., Cardeilina J.H., Boyd M.R. (1995). Mycalolides D and E, New cytotoxic macrolides from a collection of the stony coral *Tubastrea faulkneri*. J. Nat. Prod..

[50] Williams A.B., Jacobs R.S. (1993). A marine natural product, patellamide D, reverses multidrug resistance in a human leukemic cell line. Cancer Lett..

[51] Salvador-Reyes L.A., Engene N., Paul V.J., Luesch H. (2015). Targeted natural products discovery from marine cyanobacteria using combined phylogenetic and mass spectrometric evaluation. J. Nat. Prod..

[52] Hughes R.A., Moody C.J. (2007). From amino acids to heteroaromatics—thiopeptide antibiotics, nature’s heterocyclic peptides. Angew. Chem. Int. Ed..

[53] Just-Baringo X., Bruno P., Ottesen L.K., Cañedo L.M., Albericio F., Álvarez M. (2013). Total synthesis and stereochemical assignment of baringolin. Angew. Chem. Int. Ed..

[54] Butler M.S., Robertson A.A.B., Cooper M.A. (2014). Natural product and natural product derived drugs in clinical trials. Nat. Prod. Rep..

[55] Dyshlovoy S., Honecker F. (2015). Marine compounds and cancer: Where do we stand?. Mar. Drugs.

[56] Hamada Y., Kato S., Shioiri T. (1985). New methods and reagents in organic synthesis. 51. A synthesis of ascidiacyclamide, a cytotoxic cyclic peptide from ascidian—Determination of its absolute configuration. Tetrahedron Lett..

[57] Baur P., Comba P., Velmurugan G. (2022). Efficient synthesis for a wide variety of patellamide derivatives and phosphatase activity of copper-patellamide complexes. Chem. Eur. J..

[58] Koehnke J., Bent A.F., Houssen W.E., Mann G., Jaspars M., Naismith J.H. (2014). The structural biology of patellamide biosynthesis. Curr. Opin. Struct. Biol..

[59] García-Reynaga P., VanNieuwenhze M.S. (2008). A new total synthesis of patellamide A. Org. Lett..

[60] Comba P., Dovalil N., Gahan L.R., Haberhauer G., Hanson G.R., Noble C.J., Seibold B., Vadivelu P. (2012). Cu^II^ coordination chemistry of patellamide derivatives: Possible biological functions of cyclic pseudopeptides. Chem. Eur. J..

[61] Haberhauer G., Oeser T., Rominger F. (2005). A widely applicable concept for predictable induction of preferred configuration in C3-symmetric systems. Chem. Commun..

[62] Haberhauer G., Pintér Á., Oeser T., Rominger F. (2007). Synthesis and Structural Investigation of C4- and C2-Symmetric Molecular Scaffolds Based on Imidazole Peptides. Eur. J. Org. Chem..

[63] Haberhauer G., Drosdow E., Oeser T., Rominger F. (2008). Structural investigation of westiellamide analogues. Tetrahedron.

[64] Xie S., Savchenko A.I., Krenske E.H., Grange R.L., Gahan L.R., Williams C.M. (2018). Developing cyclic peptide heteroatom interchange: Synthesis and DFT modelling of a HI-ascidiacyclamide isomer. Eur. J. Org. Chem..

[65] Abbenante G., Fairlie D.P., Gahan L.R., Hanson G.R., Pierens G.K., van den Brenk A.L. (1996). Conformational control by thiazole and oxazoline rings in cyclic octapeptides of marine origin. Novel macrocyclic chair and boat conformations. J. Am. Chem. Soc..

[66] Ishida T., In Y., Shinozaki F., Doi M., Yamamoto D., Hamada Y., Shioiri T., Kamigauchi M., Sugiura M. (1995). Solution conformations of patellamides B and C, cytotoxic cyclic hexapeptides from marine tunicate, determined by NMR spectroscopy and molecular dynamics. J. Org. Chem..

[67] Ishida T., In Y., Doi M., Inoue M., Hamada Y., Shiori T. (1992). Molecular conformation of ascidiacyclamide, a cytotoxic cyclic peptide from Ascidian: X-ray analyses of its free form and solvate crystals. Biopolymers.

[68] In Y., Doi M., Inoue M., Ishida T., Hamada Y., Shioiri T. (1993). Molecular conformation of patellamide A, a cytotoxic cyclic peptide from the ascidian *Lissoclinum patella*, by x-ray crystal analysis. Chem. Pharm. Bull..

[69] In Y., Doi M., Inoue M., Ishida T., Hamada Y., Shioiri T. (1994). Patellamide A, a cytotoxic cyclic peptide from the ascidian *Lissoclinum patella*. Acta Crystallogr. Sect. C Cryst. Struct. Commun..

[70] Cusack R.M., Grøndahl L., Abbenante G., Fairlie D.P., Gahan L.R., Hanson G.R., Hambley T.W. (2000). Conformations of cyclic octapeptides and the influence of heterocyclic ring constraints upon calcium binding. J. Chem. Soc. Perkin Trans. 2.

[71] Milne B.F., Morris L.A., Jaspars M., Thompson G.S. (2002). Conformational change in the thiazole and oxazoline containing cyclic octapeptides, the patellamides. Part 2. Solvent dependent conformational change. J. Chem. Soc. Perkin Trans. 2.

[72] Haberhauer G., Rominger F. (2002). Synthesis of a new class of imidazole-based cyclic peptides. Tetrahedron Lett..

[73] Pintér Á., Haberhauer G. (2009). Synthesis of chiral threefold and sixfold functionalized macrocyclic imidazole peptides. Tetrahedron.

[74] Ishida T., Inoue M., Hamada Y., Kato S., Shioiri T. (1987). X-ray crystal structure of ascidiacyclamide, a cytotoxic cyclic peptide from ascidian. J. Chem. Soc. Chem. Commun..

[75] Endo M., Nakagawa M., Hamamoto Y., Nakanishi Y. (1983). Calvularins, a new class of cytotoxic compounds isolated from the soft coral, *Clavularia koellikeri*. J. Chem. Soc. Chem. Commun..

[76] Comba P., Dovalil N., Hanson G.R., Linti G. (2011). Synthesis and Cu II coordination chemistry of a patellamide derivative: Consequences of the change from the natural thiazole/oxazoline to the artificial imidazole heterocycles. Inorg. Chem..

[77] Comba P., Dovalil N., Haberhauer G., Kowski K., Mehrkens N., Westphal M. (2013). Copper solution chemistry of cyclic pseudo-octapeptides. Z. Anorg. Allg. Chem..

[78] Van den Brenk A.L., Fairlie D.P., Hanson G.R., Gahan L.R., Hawkins C.J., Jones A. (1994). Binding of copper(II) to the cyclic octapeptide patellamide D. Inorg. Chem..

[79] Donia M.S., Hathaway B.J., Sudek S., Haygood M.G., Rosovitz M.J., Ravel J., Schmidt E.W. (2006). Natural combinatorial peptide libraries in cyanobacterial symbionts of marine ascidians. Nat. Chem. Biol..

[80] Oueis E., Nardone B., Jaspars M., Westwood N.J., Naismith J.H. (2017). Synthesis of hybrid cyclopeptides through enzymatic macrocyclization. ChemistryOpen.

[81] Koehnke J., Bent A.F., Zollman D., Smith K., Houssen W.E., Zhu X., Mann G., Lebl T., Scharff R., Shirran S. (2013). The cyanobactin heterocyclase enzyme: A processive adenylase that operates with a defined order of reaction. Angew. Chem. Int. Ed..

[82] Houssen W.E., Bent A.F., McEwan A.R., Pieiller N., Tabudravu J., Koehnke J., Mann G., Adaba R.I., Thomas L., Hawas U.W. (2014). An efficient method for the *in vitro* production of azol(in)e-based cyclic peptides. Angew. Chem. Int. Ed..

[83] Oueis E., Jaspars M., Westwood N.J., Naismith J.H. (2016). Enzymatic macrocyclization of 1,2,3-triazole peptide mimetics. Angew. Chem. Int. Ed..

[84] Oueis E., Stevenson H., Jaspars M., Westwood N.J., Naismith J.H. (2017). Bypassing the proline/thiazoline requirement of the macrocyclase PatG. Chem. Commun..

[85] Alexandru-Crivac C.N., Umeobika C., Leikoski N., Jokela J., Rickaby K.A., Grilo A.M., Sjö P., Plowright A.T., Idress M., Siebs E. (2017). Cyclic peptide production using a macrocyclase with enhanced substrate promiscuity and relaxed recognition determinants. Chem. Commun..

[86] Wipf P. (1995). Synthetic studies of biologically active marine cyclopeptides. Chem. Rev..

[87] Comba P., Cusack R., Fairlie D.P., Gahan L.R., Hanson G.R., Kazmaier U., Ramlow A. (1998). The solution structure of a copper(II) compound of a new cyclic octapeptide by EPR spectroscopy and force field calculations. Inorg. Chem..

[88] Haberhauer G., Rominger F. (2003). Syntheses and structures of imidazole analogues of *Lissoclinum* cyclopeptides. Eur. J. Org. Chem..

[89] Morris L.A., Jaspars M., Chrystal E.J.T., Wrigley S.K., Thomas R., Nicholson N., Hayes M. (2007). A Cu^2+^ selective marine metabolite. Biodiversity: New Leads for the Pharmaceutical and Agrochemical Industries.

[90] Hamada Y., Shibata M., Shioiri T. (1985). New methods and reagents in organic synthesis. 56. Total syntheses of patellamides B and C, cytotoxic cyclic peptides from a tunicate 2. Their real structures have been determined by their syntheses. Tetrahedron Lett..

[91] You S.-L., Kelly J.W. (2003). Total synthesis of dendroamide A: Oxazole and thiazole construction using an oxodiphosphonium salt. J. Org. Chem..

[92] Morris L.A., Jaspars M., Kettenes-van den Bosch J.J., Versluis K., Heck A.J., Kelly S.M., Price N.C. (2001). Metal binding of *Lissoclinum patella* metabolites. Part 1: Patellamides A, C and ulithiacyclamide A. Tetrahedron.

[93] Morris L.A., Milne B.F., Jaspars M., Jantina Kettenes-van den Bosch J., Versluis K., Heck A.J., Kelly S.M., Price N.C. (2001). Metal binding of *Lissoclinum patella* metabolites. Part 2: Lissoclinamides 9 and 10. Tetrahedron.

[94] Latifi R., Bagherzadeh M., Milne B.F., Jaspars M., de Visser S.P. (2008). Density functional theory studies of oxygen and carbonate binding to a dicopper patellamide complex. J. Inorg. Biochem..

[95] Comba P., Gahan L.R., Haberhauer G., Hanson G.R., Noble C.J., Seibold B., van den Brenk A.L. (2008). Copper(II) Coordination chemistry of westiellamide and its imidazole, oxazole, and thiazole analogues. Chem. Eur. J..

[96] Comba P., Dovalil N., Hanson G.R., Harmer J.R., Noble C.J., Riley M.J., Seibold B. (2014). Insights into the electronic structure of Cu^II^ bound to an imidazole analogue of westiellamide. Inorg. Chem..

[97] Comba P., Eisenschmidt A., Gahan L.R., Hanson G.R., Mehrkens N., Westphal M. (2016). Dinuclear Zn II and mixed Cu^II^ –Zn^II^ complexes of artificial patellamides as phosphatase models. Dalton Trans..

[98] Bernhardt P.V., Comba P., Hambley T.W., Massoud S.S., Stebler S. (1992). Determination of solution structures of binuclear copper(II) complexes. Inorg. Chem..

[99] Van den Brenk A.L., Byriel K.A., Fairlie D.P., Gahan L.R., Hanson G.R., Hawkins C.J., Jones A., Kennard C.H.L., Moubaraki B., Murray K.S. (1994). Crystal structure and electrospray ionization mass spectrometry, electron paramagnetic resonance, and magnetic susceptibility study of [Cu_2_(ascidH_2_)(1,2-µ-CO_3_)(H_2_O)_2_]·2H_2_O, the bis(copper(II)) complex of ascidiacyclamide (ascidH_4_), a cyclic peptide isolated from the ascidian *Lissoclinum patella*. Inorg. Chem..

[100] Comba P., Gahan L.R., Hanson G.R., Maeder M., Westphal M. (2014). Carbonic anhydrase activity of dinuclear Cu II complexes with patellamide model ligands. Dalton Trans..

[101] Comba P., Eisenschmidt A., Velmurugan G. (2022).

[102] Comba P., Eisenschmidt A., Gahan L.R., Herten D.-P., Nette G., Schenk G., Seefeld M. (2017). Is Cu^II^ coordinated to patellamides inside *Prochloron* cells?. Chem. Eur. J..

[103] Comba P., Gahan L.R., Hanson G.R., Westphal M. (2012). Phosphatase reactivity of a dicopper(ii) complex of a patellamide derivative—Possible biological functions of cyclic pseudopeptides. Chem. Commun..

[104] Comba P., Eisenschmidt A., Kipper N., Schießl J. (2016). Glycosidase- and β-lactamase-like activity of dinuclear copper(II) patellamide complexes. J. Inorg. Biochem..

[105] Koehl M.A.R., Powell T.M., Dobbins E.L. Effects of algal turf on mass transport and flow microhabitats of ascidians in a coral reef lagoon. Proceedings of the 8th International Coral Reef Symposium.

[106] Kühl M., Behrendt L., Trampe E., Qvortrup K., Schreiber U., Borisov S.M., Klimant I., Larkum A.W.D. (2012). Microenvironmental ecology of the chlorophyll *b*-containing symbiotic cyanobacterium *Prochloron* in the didemnid ascidian *Lissoclinum patella*. Front. Microbiol..

[107] Kühl M., Larkum A.W.D. (2004). The Microenvironment and Photosynthetic Performance of *Prochloron* sp. in Symbiosis with Didemnid Ascidians. Symbiosis.

[108] Dionisio-Sese M.L., Ishikura M., Maruyama T., Miyachi S. (1997). UV-absorbing substances in the tunic of a colonial ascidian protect its symbiont, *Prochloron* sp., from damage by UV-B radiation. Mar. Biol..

[109] Hirose E., Ohtsuka K., Ishikura M., Maruyama T. (2004). Ultraviolet absorption in ascidian tunic and ascidian-*Prochloron* symbiosis. J. Mar. Biol. Assoc. UK.

[110] Behrendt L., Larkum A.W.D., Trampe E., Norman A., Sørensen S.J., Kühl M. (2012). Microbial diversity of biofilm communities in microniches associated with the didemnid ascidian *Lissoclinum patella*. ISME J..

[111] Komárek J., Komárková J. (2004). Taxonomic review of the cyanoprokaryotic genera *Planktothrix* and *Planktothricoides*. Fottea.

[112] Da Silva Oliveira F.A., Colares G.B., Hissa D.C., Angelim A.L., Melo V.M.M., Lotufo T.M.C. (2013). Microbial epibionts of the colonial ascidians *Didemnum galacteum* and *Cystodytes* sp.. Symbiosis.

[113] Hirose E., Turon X., López-Legentil S., Erwin P.M., Hirose M. (2012). First records of didemnid ascidians harbouring *Prochloron* from Caribbean Panama: Genetic relationships between Caribbean and Pacific photosymbionts and host ascidians. Syst. Biodivers..

[114] Donia M.S., Fricke W.F., Partensky F., Cox J., Elshahawi S.I., White J.R., Phillippy A.M., Schatz M.C., Piel J., Haygood M.G. (2011). Complex microbiome underlying secondary and primary metabolism in the tunicate-*Prochloron* symbiosis. Proc. Natl. Acad. Sci. USA.

[115] Donia M., Fricke W., Ravel J., Schmidt E. (2011). Variation in tropical reef symbiont metagenomes defined by secondary metabolism. PLoS ONE.

[116] Schmidt E.W. (2015). The secret to a successful relationship: Lasting chemistry between ascidians and their symbiotic bacteria. Invertebr. Biol..

[117] Erwin P.M., Pineda M.C., Webster N., Turon X., López-Legentil S. (2014). Down under the tunic: Bacterial biodiversity hotspots and widespread ammonia-oxidizing archaea in coral reef ascidians. ISME J..

[118] Lin Z., Torres J.P., Tianero M.D., Kwan J.C., Schmidt E.W. (2016). Origin of chemical diversity in *Prochloron*-tunicate symbiosis. Appl. Environ. Microbiol..

[119] Tianero M.D.B., Kwan J.C., Wyche T.P., Presson A.P., Koch M., Barrows L.R., Bugni T.S., Schmidt E.W. (2015). Species specificity of symbiosis and secondary metabolism in ascidians. ISME J..

[120] Lopez-Guzman M., Erwin P.M., Hirose E., López-Legentil S. (2020). Biogeography and host-specificity of cyanobacterial symbionts in colonial ascidians of the genus *Lissoclinum*. Syst. Biodivers..

[121] Kühl M., Chen M., Ralph P.J., Schreiber U., Larkum A.W.D. (2005). A niche for cyanobacteria containing chlorophyll *d*. Nature.

[122] Behrendt L., Larkum A., Norman A., Qvortrup K., Chen M., Ralph P., Sørensen S., Trampe E., Kühl M. (2011). Endolithic chlorophyll *d*-containing phototrophs. ISME J..

[123] Behrendt L., Trampe E.L., Nord N.B., Nguyen J., Kühl M., Lonco D., Nyarko A., Dhinojwala A., Hershey O.S., Barton H. (2020). Life in the dark: Far-red absorbing cyanobacteria extend photic zones deep into terrestrial caves. Environ. Microbiol..

[124] Behrendt L., Brejnrod A., Schliep M., Sørensen S.J., Larkum A.W., Kühl M. (2015). Chlorophyll *f*-driven photosynthesis in a cavernous cyanobacterium. ISME J..

[125] Trampczynska A., Küpper H., Meyer-Klaucke W., Schmidt H., Clemens S. (2010). Nicotianamine forms complexes with Zn(ii) in vivo. Metallomics.

[126] Baur P., Comba P. (2022).

[127] Paul V.J., Arthur K.E., Ritson-Williams R., Ross C., Sharp K. (2007). Chemical defenses: From compounds to communities. Biol. Bull..

[128] Flórez L.V., Biedermann P.H.W., Engl T., Kaltenpoth M. (2015). Defensive symbioses of animals with prokaryotic and eukaryotic microorganisms. Nat. Prod. Rep..

[129] Lindquist N., Hay M.E., Fenical W. (1992). Defense of ascidians and their conspicuous larvae: Adult vs. larval chemical defenses. Ecol. Monogr..

[130] Olson R.R., McPherson R. (1987). Potential vs. realized larval dispersal: Fish predation on larvae of the ascidian *Lissoclinum patella* (Gottschaldt). J. Exp. Mar. Bio. Ecol..

[131] Schenk G., Mitić N., Hanson G.R., Comba P. (2013). Purple acid phosphatase: A journey into the function and mechanism of a colorful enzyme. Coord. Chem. Rev..

[132] Geier B., Sogin E.M., Michellod D., Janda M., Kompauer M., Spengler B., Dubilier N., Liebeke M. (2020). Spatial metabolomics of in situ host–microbe interactions at the micrometre scale. Nat. Microbiol..

[133] Behrendt L., Salek M.M., Trampe E.L., Fernandez V.I., Lee K.S., Kühl M., Stocker R. (2020). PhenoChip: A single-cell phenomic platform for high-throughput photophysiological analyses of microalgae. Sci. Adv..

